# Evaluation of serum miRNAs expression in frail and robust subjects undergoing multicomponent exercise protocol (VIVIFRAIL)

**DOI:** 10.1186/s12967-023-03911-3

**Published:** 2023-02-02

**Authors:** Simone Agostini, Roberta Mancuso, Lorenzo Agostino Citterio, Gabriela Alexandra Mihali, Beatrice Arosio, Mario Clerici

**Affiliations:** 1 Laboratory of Molecular Medicine and Biotechnologies, IRCCS Fondazione Don Carlo Gnocchi ONLUS, Piazza Morandi 3, 20100 Milan, Italy; 2 Geriatic Unit, Fondazione IRCCS Ca’ Granda Ospedale Maggiore Policlinico, Milan, Italy; 3 Department of Clinical Sciences and Community Health, University of Milan, Milan, Italy; 4 Department of Pathophysiology and Transplantation, University of Milan, Milan, Italy

**Keywords:** microRNA, Sarcopenia, Frailty, Rehabilitation, Physical Activity, Biomarkers

## Abstract

**Background:**

Frailty, defined as physical performance impairment, is a common condition in older adults and can anticipate the development of sarcopenia, a geriatric syndrome characterized by loss of muscle strength and mass. microRNAs (miRNAs) are short molecules of RNA endowed with the ability to modulate gene expression; miRNAs are present in serum and are considered potential biomarkers for several diseases. Serum concentration of miR-451a, miR-93-5p, miR-155-5p, miR-421-3p, miR-425-5p, miR-495-3p and miR-744-5p was recently shown to be altered in sarcopenic patients.

**Methods:**

We verified if a particular miRNAs pattern could be detected in frailty as well by analyzing these molecules in 50 frail and 136 robust subjects. Additionally, a subgroup of these subjects (15 frail and 30 robust) underwent a 12-week program based on a multicomponent exercise protocol (VIVIFRAIL) consisting of resistance training, gait retraining, and balance training. After the program, serum miRNAs concentration was measured again, to verify whether the physical activity had an effect on their concentration. Moreover, clinical characteristics and indicators of physical performance of all subjects were compared before and after intervention to verify the effect of the VIVIFRAIL program.

**Results:**

At the end of the multicomponent exercise program, Short Physical Performance Battery (SPPB) score as well right and left handgrip (p < 0.05) were significantly increased in frail subjects; right and left handgrip significantly were increased also in robust subjects (p < 0.05). Interestingly, the variation of SPPB was significantly higher in frail compared to robust subjects (p < 0.0001). Moreover, at the end of the program, in frail compared to robust subjects: miR-451a serum concentration was significantly increased (frail: 6.59 × 10^4^; 1.12 × 10^4^–2.5 × 10^5^ c/ng; robust: 2.31 × 10^4^; 1.94 × 10^3^–2.01 × 10^5^ c/ng) (p < 0.05); and 2) miR-93-5p and miR-495-3p serum concentration was reduced, whereas that of miR-155-5p was significantly increased (p < 0.05 in both cases). Serum concentration of miR-93-5p and miR-495-3p was decreased, and that of miR-155-5p was increased at the end of the program in robust subjects alone, statistical significance being reached for miR-93-5p alone (p = 0.02).

**Conclusion:**

These results suggest that serum miR-451a should be investigated as a potential biomarker for frailty and show that the VIVIFRAIL multicomponent program modulates circulatory miRNAs expression, at least in older adults.

## Background

The higher clinical complexity that features older adults is well represented by the concept of frailty, a condition characterized by increased vulnerability to stressors and reduced homeostatic reserves [1]. Physical decline is considered the cardinal sign of frailty [2, 3]; indeed, aging is typically characterized by muscle wasting that progressively causes disability, loss of muscle function and of self-sufficiency in older adults. This phenomenon is called sarcopenia, a term that describes the age-related loss of muscle mass and muscle strength and/or function (i.e., dynapenia) [4–8]. The etiology of sarcopenia is multifactorial, involving many biological mechanisms [9, 10], like neuromuscular junction dysfunction, reduced satellite cell number/function, intramuscular adipose tissue infiltration [11] as well as chronic inflammation [12]. It has been proposed that sarcopenia may be the biological substrate for the development of physical frailty [13, 14]. However, the causal relationship between the two manifestations remains largely unknown.

Several proteins and biomolecules involved in inflammation and in oxidative stress are hypothesized to play a pathogenetic role in frailty and sarcopenia [15]. Among these biomolecules, microRNAs (miRNAs) are involved in various pathways and cellular mechanisms that are potentially associated with sarcopenia, including satellite cell regulation [16, 17] and the functionality of muscle fibers and mitochondria [18]. Notably, miRNAs have also repeatedly been associated with chronic conditions in which sarcopenia is more frequently observed, such as neurodegenerative pathologies [reviewed by 19].

miRNAs are short single-stranded RNA molecules (about 22 nucleotides) that are usually coded within introns. miRNAs recognize complementary patterns mainly in the 3′ untranslated region (3′-UTR) of target mRNAs and regulate their expression by degradation or translation blockage, acting as critical post-transcriptional mediators of cell function [20–22]. miRNAs also play a crucial role in diseases, as their ability to regulate the expression of specific genes results in the control and modulation of the activity of multiple biological pathways [reviewed by 22].

We have recently shown that serum miR-451a concentration is significantly increased in severe sarcopenia; we also observed that serum concentration of this miRNA, as well as that of miR-93-5p, miR-155-5p, miR-421-3p, miR-425-5p, miR-495-3p and miR-744-5p, is modulated by rehabilitation in severe sarcopenic patients [23]. On the basis of that finding, we analyzed whether these miRNAs are deregulated in frail subjects as well in comparison with robust subjects, and verified if an intervention program based on a multicomponent exercise protocol could modulate their expression.

## Material and methods

### Patients and controls

One-hundred-eighty-six individuals were included in the study: 50 (16 males and 34 females) frail subjects and 136 (43 males and 93 females) robust subjects (controls). All subjects were recruited by the Rehabilitative Unit of the IRCCS Santa Maria Nascente, Fondazione Don Gnocchi ONLUS, and by the Fondazione IRCCS Ca’ Granda, Ospedale Maggiore Policlinico, both in Milan, Italy. SPPB score (Short Physical Performance Battery) of the frail individuals ranged between 3 and 9, and was > 9 in robust subjects [24, 25].

A subgroup of enrolled subjects (15 frail and 30 robust subjects) underwent a program of physical activity at home. This intervention program of 12 weeks was based on multicomponent exercise protocol (VIVIFRAIL©) [26] consisting of resistance training, gait retraining, and balance training. Different functional capacity levels were determined based on the scores obtained from SPPB and the 6 m gait velocity test, with each leading to the recommendation of a certain customized multicomponent physical exercise program. Programs include arm and leg strength and power exercises, balance and coordination to prevent falls, flexibility and cardiovascular endurance exercises [27]. Before as well after exercise protocols, all patients and controls were characterized for: SPPB [28], Activity of Daily Living (ADL) [29], Mini-Mental State Examination (MMSE) [30], Clock Drawing test (CDT) [31] and Geriatric Depression Scale (GDS) [32]. Inclusion criteria were: age > 60 years, sufficient cognitive abilities to both provide informed consent to the study and to participate and follow the multicomponent exercise (MMSE ≥ 24), and ability to walk in autonomy. Exclusion criteria were: localized loss of strength and aphasia due to severe stroke, severe impairment of motor skills, steroid therapy, and concomitant diagnosis of neoplastic or neurodegenerative diseases.

The study was performed in accordance with the Declaration of Helsinki; an informed consent was signed by all the participants according to a protocol approved by the ethics committee of the IRCCS Fondazione Don Carlo Gnocchi ONLUS (n#9_04/04/2018).

### Serum miRNAs extraction and cDNA reverse transcription

Serum was obtained from blood by centrifugation (2000 *g* × 10’ at room temperature). Absence of hemolysis was evaluated by visual inspection and by spectrophotometric measurement of hemoglobin absorbance at 414 nm [33]. miRNAs were semi-automatically extracted from 200 µl of serum using a column-based kit (miRNeasy serum/plasma kit, Qiagen GmbH, Hilden, Germany) by robotic workstation (Qiacube, Qiagen), according to manufacturer’s instruction. miRNAs were quantified by Qubit microRNA assay kit (Thermo Fisher, Foster City, CA, US) with a Qubit 3.0 Fluorometer (Thermo Fisher), according to manufacturer’s recommendation. Equal concentration of miRNAs was retro-transcribed in cDNA (miRCURY LNA RT kit, Qiagen) with the following protocol: 60’ at 42 °C, heat-inactivation of reverse transcriptase enzyme for 5’ at 95 °C, and a hold at 4 °C. All the variables involved in the procedure were kept consistent throughout the study to avoid variations due to sample differences and handling.

### miRNAs quantification by droplet digital PCR (ddPCR)

miR-93-5p, miR-155-5p, miR-421-3p, miR-425-5p, miR-451a, miR-495-3p and miR-744-5p were quantified by droplet digital PCR (ddPCR QX200, Bio-Rad, Hercules, CA, US). Briefly, 3 µl cDNA (1:25), mixed with specific LNA™-primers (Qiagen) and ddPCR EvaGreen Supermix (Bio-Rad), were emulsified with droplet generator oil (Bio-Rad) using a QX200 droplet generator, according to manufacturer’s instruction. A no template control and a negative control for each reverse transcription reaction were included in every assay to check for lack on non-specific amplification. Droplets were transferred into a plate, heat-sealed with a piercable sealing foil sheet (PX1, PCR plate sealer, Bio-Rad), and followed by end-point amplification (10’ at 95 °C; 40 cycles: 94 °C for 30’’, 58 °C for 60’’, 10’ at 98 °C; hold at 4 °C). Plates were then loaded into the Droplet Reader (QX200 droplet reader, Bio-Rad). Each well was queried for fluorescence to determine the quantity of positive events (droplets), and the results were displayed as dot plots. Samples resulted in less than two positive droplets are considered negative [34]. The miRNAs concentration was expressed as copies/ng (c/ng) of extracted RNA. QuantaSoft software, version 1.7.4.0917 (BioRad) and QX software, version 1.2 (Bio-Rad) were used to quantified copies of miRNAs. Thresholds were determined manually for each experiment, according to the negative controls, which included a no template control.

### Statistical analysis

Normally distributed data were summarized as mean ± standard deviation, whereas not-normally distributed data were summarized as median and interquartile range (IQR). Categorical data were compared using Pearson’s Chi squared test. Demographic and clinical data—MMSE excluded—were normally distributed data and the comparison between frail and robust subjects was analyzed using student’s t-test, whereas the comparison before and after the multicomponent exercise activity inside each group was analyzed using paired sample t-test. MMSE and biological data (miRNAs) were not-normally distributed and the comparison between frail and robust subjects was analyzed using Mann–Whitney U test, whereas the comparison before and after the multicomponent exercise activity inside each group was analyzed using Wilcoxon rank-sum test. Correlations were tested with Spearmen’s correlation coefficient. The p-values corresponding to ≤ 0.05 were described as statistically significant in the text. Statistical analyses were performed using commercial software (MedCalc Statistical Software, version 14.10.2, Ostend, Belgium).

## Results

### Clinical characteristics

Fifty frail subjects and 136 robust subjects were enrolled in the study. According to the original study plan, all 50 frail subjects and 50 robust controls were supposed to undergo the VIVIFRAIL multicomponent exercise program, but, due to COVID pandemic, this had to be modified. Thus, 15 frail and 30 robust subjects were able to complete the VIVIFRAIL program. Demographic and clinical characteristics of the study population are summarized in Table 1. Age, gender and MMSE were similar in the two groups. As per definition, SPPB was significantly lower in frail subjects compared to robust ones, before (8; 7–9 *vs.* 11; 11–12) as well after intervention (10; 9–11 *vs.* 12; 12–12) (p < 0.0001 for both comparisons). Similarly, right and left handgrip was significantly lower in frail subjects compared to robust before (right: p = 0.015; left: p = 0.03) and after intervention (right: p = 0.03; left p = 0.01). Importantly, SPPB as well as right and left handgrip significantly improved after intervention both in frail subjects (SPPB: p < 0.0001; right handgrip: p = 0.03; left handgrip: p = 0.007) and in robust subjects (p < 0.0005 for all the parameters). Interestingly, the variation of SPPB was significantly higher in frail subjects (2.1 ± 0.99) compared to robust subjects (0.55 ± 0.76) (p < 0.0001), whereas no statistical differences were observed between the two groups regarding the variation of left and/or right handgrip.

**Table 1:** Demographic and clinical characteristics of frail subjects and of age-and-sex-matched robust subjects

	Frail subjects	Robust subjects
N	50	136
Gender (F:M)	34:16	93:43
Age, years	82.42 ± 5.32	81.93 ± 6.15
MMSE pre-intervention	28.00 ± 1.64	28.86 ± 1.31
MMSE post-intervention	28.85 ± 1.53	29.40 ± 0.54
SPPB pre-intervention	8 7–9	11* 11–12
SPPB post- intervention	10 9–11¶	12 12–12*#
ADL pre-intervention	5.7 ± 0.4	5.6 ± 0.5**
ADL post-intervention	5.7 ± 0.4	5.6 ± 0.5**
Right Handgrip pre- intervention	19.39 ± 7.20	23.18 ± 8.45**
Right Handgrip post-rehabilitation	20.68 ± 5.97¶	26.80 ± 9.45**#
Left Handgrip pre- intervention	17.90 ± 7.65¶#	21.12 ± 7.46**
Left Handgrip post- intervention	19.41 ± 5.72¶	25.36 ± 7.63**#
CDT pre-intervention	2.95 ± 1.91	3.86 ± 1.50*
CDT post-intervention	3.22 ± 2.22	4.31 ± 1.28
GDS pre-intervention	5.47 ± 5.06	3.93 ± 3.77
GDS post-intervention	4.44 ± 4.56	2.66 ± 2.50

Age, MMSE, handgrip, ADL, CDT and GDS: mean ± standard deviation; SPPB: median and Interquartile range. *ADL* Activity of daily living, *CDT* Clock Drawing Test, *GDS* Geriatric Depression Scale, *MMSE* mini mental state evaluation, *SPPB* Short Physical Performance Battery

^*^p < 0.005 Frail subjects *vs.* robust subjects

^**^p < 0.05 Frail subjects *vs.* robust subjects

^¶^p < 0.05 Frail subjects before *vs.* after intervention

^#^p < 0.05 Robust subjects before *vs.* after intervention

**Table 2:** Serum miRNAs expression in frail subjects and in robust subjects before and after intervention.

	Frail subjects before intervention	Frail subjects after intervention	Robust subjects before intervention	Robust subjects after intervention
miR-93-5p (copies/ng)	1.04 × 10^3^; 1.33 × 10^2^–5.83 × 10^3^	2.67 × 10^2^; 0.00–6.23 × 10^2^	6.17 × 10^2^; 3.25 × 10^1^–6.79 × 10^3^	3.25 × 10^2^; 0.00–9.00 × 10^2^*
miR-155-5p (copies/ng)	0.00; 0.00–1.39 × 10^2^	6.41 × 10^1^; 0.00–1.90 × 10^2^	0.00; 0.00–1.56 × 10^2^	4.48 × 10^1^; 0.00–1.50 × 10^2^^
miR-421-3p (copies/ng)	0.00; 0.00–3.03 × 10^2^	0.00; 0.00–2.31 × 10^2^	0.00; 0.00–2.15 × 10^2^	0.00; 0.00–5.58 × 10^2^
miR-425-5p (copies/ng)	5.25 × 10^4^; 0.00–2.54 × 10^5^	2.67 × 10^2^; 0.00–6.68 × 10^2^	6.33 × 10^2^; 0.00–2.01 × 10^5^	0.00; 0.00–4.50 × 10^2^
miR-451a (copies/ng)	6.59 × 10^4^; 1.12 × 10^4^–2.54 × 10^5^	6.56 × 10^3^; 3.03 × 10^3^–1.78 × 10^4^	2.31 × 10^4^; 1.94 × 10^3^–2.01 × 10^5^	1.20 × 10^4^; 3.23 × 10^3^-3.72 × 10^4^
miR-495-3p (copies/ng)	4.39 × 10^2^; 2.20 × 10^2^–1.58 × 10^3^	1.17 × 10^2^; 0.00–4.65 × 10^2#^	3.20 × 10^2^; 1.83 × 10^2^–1.25 × 10^3^	3.07 × 10^2^; 0.00–1.44 × 10^3^*
miR-744-5p (copies/ng)	0.00; 0.00–1.08 × 10^2^	0.00; 0.00–1.00 × 10^2^	0.00; 0.00–1.56 × 10^2^	0.00; 0.00–1.17 × 10^2^

Data are reported as median and interquartile range.

*p = 0.02 Robust subjects before *vs.* after intervention.

#p = 0.001 Frail subjects before *vs.* after intervention.

^p = 0.05 Robust subjects before *vs.* after intervention.

### Serum concentration of the miRNAs of interest by ddPCR

**Fig. 1:**
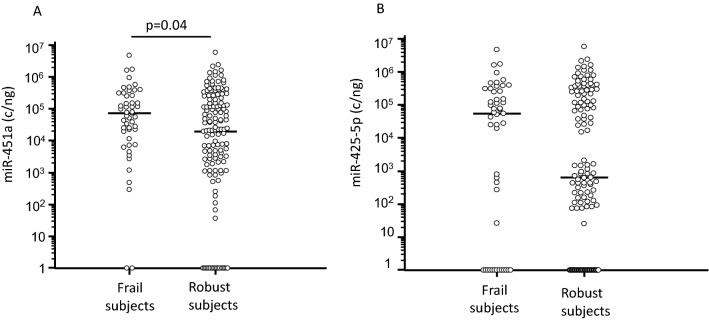
miR-451a and miR-425-5p serum concentration in frail and in age-and sex-matched robust subjects. miR-451a (Panel **A**) and miR-425-5p (Panel** B**) serum concentration in frail subjects and in robust subjects. Horizontal bars indicate median

Analysis of miRNAs serum concentration by ddPCR in frail and in robust subjects before intervention showed that miR-451a was significantly augmented in frail subjects (6.59 × 10^4^; 1.12 × 10^4^–2.5 × 10^5^ c/ng) compared to robust (2.31 × 10^4^; 1.94 × 10^3^–2.01 × 10^5^ c/ng; p = 0.04) (Fig. 1A). Concentration of miR-425-5p was higher as well in serum of frail subjects (5.25 × 10^4^; 0.00–2.54 × 10^5^ c/ng) compared to robust (6.33 × 10^3^; 0.00–2.01 × 10^5^ c/ng), even if this difference approached but did not reach statistical significance (Fig. 1B). In robust subjects, miR-425-5p serum concentration appeared to cluster into two different zones: high and low. Analyses performed on demographic and clinical (i.e. SPPB value, handgrip, sex, age) parameters did not reveal any differences between robust subjects in whom miR-425-5p concentration was either high or low.

**Fig. 2:**
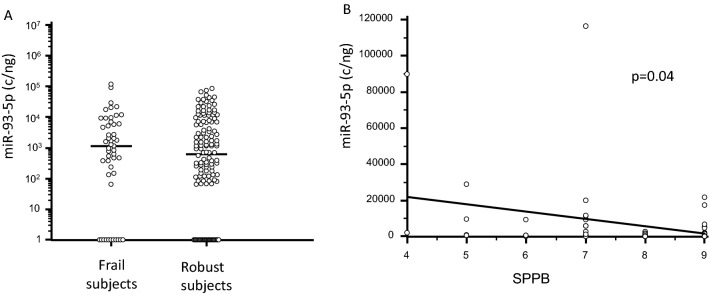
miR-93-5p serum concentration in frail and in age-and sex-matched robust subjects. Panel** A** miR-93-5p serum concentration in frail subjects and in robust subjects. Horizontal bars indicate median. Panel **B** Correlation between miR-93-5p serum concentration and SPPB score in frail subjects. Regression line is represented in the figure

**Fig. 3:**
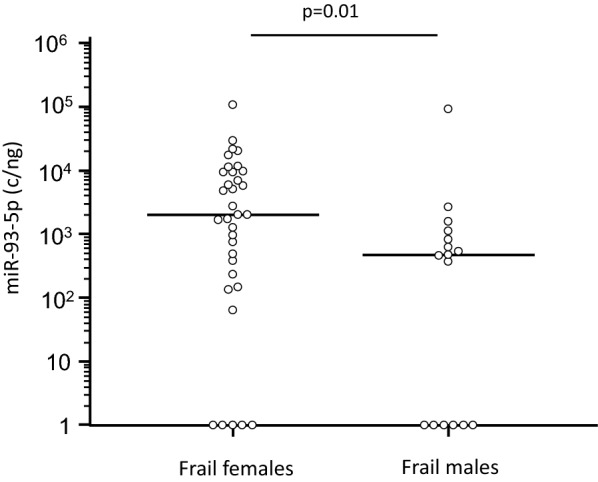
miR-93-5p serum concentration in frail subjects classified based on gender. Horizontal bars indicate median

Serum concentration of miR-93-5p was also increased, although not significantly, in frail subjects (Fig. 2A); in these individuals alone miR-93-5p concentration was negatively correlated with SPPB scores (p = 0.04, Fig. 2B). In the frail subjects alone miR-93-5p was also significantly augmented in females (1.98 × 10^3^; 2.33 × 10^2^–9.17 × 10^4^ c/ng) compared to males (4.64 × 10^2^; 0.00–9.74 × 10^3^ c/ng; p = 0.01) (Fig. 3), and was negatively correlated with right handgrip (p = 0.016).

No differences were observed regarding the other miRNAs (miR-155-5p, miR-421-3p, miR-495-3p and miR-744-5p) (Table 2) we analyzed.

### Effect of intervention on serum miRNAs

We finally verified whether an intervention program of physical activity based on a multicomponent exercise protocol (VIVIFRAIL) consisting of resistance training, gait retraining, and balance training could have an effect on serum concentration of miRNAs.

miR-93-5p and miR-495-3p serum decreased at the end of the program both in frail and in robust subjects; the variation was statistically significant for both miRNAs in robust subjects (p = 0.02 for both), whereas it was statistically significant for miR-495-3p alone (p = 0.02) in frail subjects.

An opposite effect was observed for miR-155-5p, whose serum concentration increased at the end of the intervention program; the difference reached statistical significance in robust subjects (p = 0.04). Intervention did not modulate serum concentration of the other miRNAs (miR-421-3p, miR-425-5p, miR-451a and miR-744-5p) either in frail or in robust individuals (Table 2).

## Discussion

In the present work we analyzed the serum miRNA concentration in frail and robust subjects who underwent a multicomponent exercise program; results showed that: (1) miR-451a is more expressed in frail compared to robust subjects; and (2) the multicomponent exercise program results on a differential effect on miRNAs expression, as it increased miR-155-5p and reduced miR-93-5p and miR-495-3p.

The age-related decline of muscle activity (loss of mass and strength) is an overlapping aspect between sarcopenia (a complex disease in which several factors—genetics, environmental, inflammation, hormones, neuromuscular degeneration) and frailty, a state of increased vulnerability to stress due decreased physiological reserve in multiple functions [35, 36]. Because in western countries life expectancy constantly increases, age-related diseases, are a major concern for public health. For this reason, the definition of biomolecules that could be used as biomarkers of physical frailty and/or possible risk of developing sarcopenia, would be extremely important.

In our previous work, serum concentration of miR-451a was found to be significantly increased in severe sarcopenic patients compared to robust subjects, suggesting this miRNA as a possible biomarker of the disease [23]. In the present work we found that serum concentration of this same miRNA is increased as well in physical frail subjects: individuals with a SPPB score lower than robust subjects but not (yet) suffering from sarcopenia.

miR-451a is coded by a gene localized at chromosome 17 (17q11.2) [37], is mainly expressed in skeletal muscle, and is known to play a key role in several human tumors [38–41]. Interestingly, miR-451a expression was observed to be increased in muscles of powerlifter [42] and of people that are low-responders to resistance exercise training [43]. miR-451a concentration in skeletal muscle was also shown to be inversely correlated to age in rhesus macaques [44], whereas its plasma concentration was inversely correlated with whole body muscle mass in middle aged men [42]. Notably, both findings indicate a possible role played by this miRNA in the age-associated decline of muscle mass and function characteristic of frailty and sarcopenia. miR-451a is also present in serum, where it can be found either inside vesicles or as non-vesicle associated protein (miRNA-protein, or miRNA-lipid/lipoprotein complexes) [40, 45–48]. Importantly, when inside vesicles miR-451a miRNA regulates innate immune response, as it was shown to suppress the production of pro-inflammatory cytokines after influenza vaccine [45] and, at least in vitro, even in the course of influenza A infection [49]. To the best of our knowledge, though, the only results suggesting an involvement of miR-451a in sarcopenia are the ones we have recently published [23], and no data are available on the possible relationship between this miRNA and frailty. Results herein indicating that plasma concentration of miR-451a is significantly increased in physical frailty suggests that this miRNA is a precocious and sensible biomarker of age-related muscle alterations that can be analyzed in easy-to-collect samples obtained with a standard venipuncture.

miR-425-3p and miR-93-5p were more expressed in serum of frail subjects as well, although without reaching statistical significance. To note, in these individuals the concentration of miR-93-5p negatively correlated with SPPB score. Thus, miR-93-5p serum concentration in physical frail subjects is very similar to that of robust individuals, and its concentration increases with the worsening of the impairment. Alteration in serum concentration of miR-425-3p (3p21.31) and miR-93-5p (7q22.1) were observed to be associated with multiple human tumors [50–53]. The mouse mmu-miR-425-3p was also found to be expressed in animal muscle tissue and to be a possible biomarker for viral myocarditis, suggesting a role for this miRNA in muscle-related pathologies. Plasma concentration of miR-93-5p, on the other hand, was recently shown to be reduced in sarcopenic patients [54]. These data are in apparent contrast with ours; this apparent discrepancy can be explained as follows: (1) we studied physical frail individuals whereas He analyzed sarcopenic patients; and (2) we analyzed miR-93-5p serum concentration by ddPCR, which quantifies in an absolute way (copies/ng) miRNA concentration, whereas He measured miR-93-5p in plasma using traditional qPCR, a method in which miRNA concentration is relative to a synthetic reference miRNA. Notably, miR-93-5p was shown to be augmented also in serum, bone tissue and bone cells of patients suffering from osteoporosis, in whom miR-93-5p concentration was correlated with bone mass density [55]. Nevertheless, studies in larger groups, analyzing miRNAs expression in serum and in plasma, and possibly including different ethnicity, are necessary to clarify this discrepancy.

All the individuals enrolled in the study underwent an intervention program based on physical activity at home. Importantly, this activity has an important positive and beneficial effects in both groups, with a significant improvement of physical condition as measured by SPPB and handgrip, in particular for frail subjects, in accordance with previous papers [reviewed by 56], indicating that this multicomponent program is a useful strategy to improve physical performance, at least in frailty.

Physical activity modulated serum expression of miR-93-5p, miR-495-3p and miR-155-5p both in frail and robust subjects. In particular: miR-495-3p and miR-93-5p were robustly down-regulated whereas miR-155-5p was up-regulated by intervention in in both groups. A very limited amount of data is available on the effect of program of intervention on circulatory miRNAs expression, and these data usually stem from analyses performed in athletes or in young people [i.e. 57, 58]. Scarce results are available on the effect of acute or intense exercise on circulatory miRNAs expression in older adults (i.e. 57, 59]. We have recently shown that rehabilitation has an effect on the expression of miRNAs that are related to SNAP-25 and IL-17 expression in severe sarcopenic patients [23, 60]. To the best of our knowledge, these are the first results showing the intervention has a significant effect on the expression of miRNA detectable in serum and possibly involved in muscle metabolism in physical frail subjects. Notably, results herein suggest that miRNAs expression is modulated by physical activity in older adults. As these proteins target several biological processes, modulation of their expression might regulate diverse mechanisms that contribute to the pathophysiology of the frailty. Although the multicomponent exercise program was similar for frail and robust subjects, and although the demographic variables were comparable between the two groups, we cannot exclude that other factors, including drugs, diet, comorbidities, etc., could have influenced the miRNAs expression.

## Conclusions

To summarize, our results indicate that miR-451a expression is altered not only in sarcopenia, but in frailty as well, and that miR-93-5p, miR-155-5p and miR-495-3p can be modulated by a program of physical intervention. These data suggest that miRNAs serum concentration might be used as a biomarker in the diagnosis, the prognosis and the evaluation of physical and rehabilitative protocols in age-related muscle degenerative pathologies.

## Data Availability

The dataset generated and/or analyzed during the current study are not publicly available due to privacy or ethical restriction but are available from the corresponding author on reasonable request.

## Abbreviations

ADL: Activity of daily living
CDT: Clock Drawing test
ddPCR: Droplet digital polymerase chain reaction
GDS: Geriatric Depression Scale
IADL: Instrument activity of daily living
IL-17: Interleukin-17
IQR: Interquartile range
miRNA: MicroRNA
MMSE: Mini-Mental State Examination
PCR: Polymerase chain reaction
SNAP25: SNARE synaptosomal-associated protein of 25 kDa
SPPB: Short Physical Performance Battery
